# Utility of glass slide morphology (GSM) and whole slide image (WSI) in the diagnosis of acute leukemia (AL) by types

**DOI:** 10.3332/ecancer.2024.1790

**Published:** 2024-10-31

**Authors:** Hamisa Iddy, Ahlam Nasser, Ally Hussein, Anna Schuh, Clara Chamba

**Affiliations:** 1 Ocean Road Cancer Institute (ORCI), Dar es salaam 11101, Tanzania; 2 Muhimbili University of Health and Allied Sciences (MUHAS), Dar es salaam 11103, Tanzania; 3 Tanzania Field Epidemiology and Laboratory Training Program, Dar es Salaam 11101, Tanzania; 4 University of Oxford, Oxford OX37DL, UK

**Keywords:** acute leukemia, glass slide morphology, whole slide image, flow cytometry, sensitivity, specificity, Tanzania

## Abstract

Acute leukemia (AL) is a diverse group of hematological malignancies characterised by the accumulation of immature blast cells in the bone marrow. Accurate classification into acute myeloid leukemia (AML) or acute lymphoblastic leukemia (ALL) is essential for treatment and prognosis. This study aimed to assess the performance of glass slide morphology (GSM) using a light microscope versus whole slide imaging (WSI) in diagnosing and classifying AL, using flow cytometry as the gold standard test. Peripheral smears and bone marrow aspirates from 97 patients suspected of AL were stained with Romanowsky stain and reviewed by a single hematologist. For GSM, the hematologist was provided with a single slide, which was to be evaluated under a light microscope. For WSI, the Alexapath mobile scanner (ADA1) was used to scan the slides for review by the hematologist. Patient identification was concealed from the interpreting hematologist, and an interval of 2 weeks was set between the review of GSM and WSI of the same patient. The sensitivity and specificity of GSM and WSI were compared to the results of flow cytometry. Out of the 97 patients suspected to have AL, 47 were confirmed to have AL by flow cytometry. Among these, 19 (40.4%) were diagnosed with AML and 28 (59.6%) with ALL. GSM demonstrated high sensitivity (89.4%) and specificity (90.0%) for diagnosing AL, but lower sensitivity in distinguishing AML (57.9%) from ALL (75.0%). Similarly, WSI exhibited a reasonable sensitivity (80.9%) and high specificity (98.0%) for diagnosing AL, but lower sensitivity in differentiating AML (57.9%) and ALL (46.4%). GSM and WSI are reasonable and acceptable techniques for accurately screening AL cases and accelerating referral to tertiary centers of excellence.

## Introduction

Acute leukemia (AL) is a heterogeneous group of malignancies affecting all age groups with an average annual incidence rate of 4–7 per 100,000 population [1]. It is characterised by the accumulation of immature blast cells in the bone marrow, which replace normal marrow tissue, including hemopoietic precursor cells [2]. Based on precursor cells of origin, AL can be broadly classified into acute myeloid leukemia (AML) and acute lymphoblastic leukemia (ALL). With advancements in treatment, it has become increasingly important to accurately classify the type of AL based on morphology, cytochemistry, immunophenotyping, cytogenetic and molecular genetics studies [3]. This categorization bears prognostic and therapeutic implications, allowing individualization of the type and intensity of treatment according to the leukemia type. Additionally, immunophenotyping of surface antigens expressed by the leukemia cells using flow cytometry is considered the gold standard method for accurately classifying AL as either AML or ALL. However, due to high costs associated with flow cytometry and other sophisticated investigations such as cytogenetic and molecular genetics studies, these tests remain unavailable and inaccessible to the majority of patients in many resource-limited settings, including Tanzania. As a result, morphologic assessment remains the mainstay of diagnosis.

Over the years, morphologic assessment of leukemia blasts has been done using traditional glass slide morphology (GSM) examination under the microscope. With the advancement of technology, whole slide imaging (WSI) and digital pathology have been increasingly used in clinical practice. WSI may be used as a substitute for the traditional GSM examination in areas with limited trained personnel to accurately make the diagnosis of AL. This is particularly important in sub-Sahara African countries including Tanzania where there is a limited number of pathologists and hematologists. The average number of pathologists per head of population in sub-Saharan Africa is 1/1,000,000 compared to 1 pathologist to 15–20,000 in the US and UK [4]. To date, in Tanzania, there are a total number of 40 hematologists serving a population of almost 60 million people [5]. The majority of these are concentrated in the capital city of Dar es salaam where the National Referral Hospital that provides specialist care is located. As a result, there are significant delays in the diagnosis of hematologic disorders including AL. A potential solution to bridge the gap of adequately trained personnel is the use of WSI where technical health care personnel from peripheral hospitals scan and send digital whole slide images of the peripheral blood smears for morphological review by specialist hematologists at the zonal or national hospitals. This will allow faster diagnosis and early referral to the specialised center for definitive diagnosis. This is even more crucial in the setting of AL where timely diagnosis is critical for patients’ survival. Therefore, this study aimed to evaluate the performance of GSM and WSI in diagnosing AL and its types using flow cytometry as the gold standard test.

## Methods

### Study design and settings and study population

This was a hospital-based cross sectional study conducted at Muhimbili National Hospital (MNH) in Dar es Salaam from January to May 2019. The recruitment point for this study was the bone marrow aspiration room, where all adult and pediatric patients who had been previously reviewed by a hematologist and were suspected of having AL based on clinical presentation, complete blood counts and peripheral smears were enrolled. The exclusion criteria consisted of previously diagnosed AL, relapsed AL and cases where bone marrow aspiration resulted in a dry tap.

### Sample collection and processing

All bone marrow samples of the recruited patients were processed for GSM and WSI. Additionally, for each of the consenting patients undergoing a bone marrow aspiration, their previous complete blood count and smear results were reviewed to determine if the patient had peripheral circulating blasts for flow cytometry. For patients with cytopenia and no circulating blasts, a bone marrow aspirate sample was used for flow cytometry.

### Morphologic examination of the smears

Bone marrow aspirate smears were evaluated and reviewed by one experienced hematologist after staining with Romanowsky stain. For GSM, the hematologist was provided with a single slide which was to be evaluated under a light microscope. For WSI, the Alexapath mobile scanner (ADA1) was used to scan the slides, acquiring a total of 20 images per patient slide for review by the hematologist. Patient identification was concealed from the interpreting hematologist, and an interval of 2 weeks was set between the review of GSM and WSI of the same patient. The type of AL was classified into AML or ALL according to FAB classification.

### Immunophenotyping by flow cytometry

Immunophenotyping was done using multiparametric, eight-colour flow cytometer (Beckman Coulter, FACS Canto II). Panels of monoclonal antibodies from the European Group for the Immunological Characterization of Leukemia criteria were used. Cell suspensions were stained with multiple panels of three monoclonal antibodies and a two-step strategy was used. The samples were then labeled with fluorescein isothiocyanate, Phycoerythrin and Peridin Chlorophyll Protein Complex. Cell suspensions were also stained with panels of identically conjugated isotype controls for the antibodies of each panel. Leukemic samples were considered positive for a particular antigen if 20% or more of leukemic cells reacted with a particular monoclonal antibody. The panels of antibodies included the following: CD34, HLA-DR, CD117, CD13, CD14, CD33, CD19, CD10, CD20, CD2, CD3, CD5, CD7, CD45 and CD64.

A case was considered B ALL if it expressed (CD10+, CD19+, CD20+, CD34+ and HLADR+). The T-cell ALL were considered when the markers for T-cell were positive (CD2+, CD3+, CD5+, CD7+ and CD34+). Myeloid cases were considered AML if expressed progenitor antigens (HLA-DR, CD34 and CD117), as well as myeloid antigens (CD13, CD33, CD14 and CD64).

### Statistical analysis

We conducted an analysis to assess the effectiveness of GSM and WSI in diagnosing AL by types, specifically AML and ALL. We evaluated the diagnostic performance of GSM and WSI compared to flow cytometry results, considered the gold standard. Sensitivity, specificity, positive predictive value (PPV), negative predictive value (NPV) and overall accuracy were calculated and expressed in percentages.

These measures were determined using the following formulas:

Sensitivity (%) = (True positives (TP) / (TP + False negatives (FN))) × 100

Specificity (%) = (True negatives (TN) / (TN + False positives (FP))) × 100

PPV (%) = (TP / (TP + FP)) × 100

NPV (%) = (TN / (TN + FN)) × 100

Accuracy (%) = ((TP + TN) / Total Samples) × 100

TP represents cases where GSM or WSI correctly identified the presence of AL in the sample. FN occurs when GSM or WSI incorrectly indicates the absence of AL when it was actually present. TN are cases where GSM or WSI correctly identified the absence of AL. FP occurs when GSM or WSI incorrectly indicates the presence of AL when it was actually absent.

The analysis was performed using epi info 7.2 statistical software (CDC, USA), and descriptive statistics were used to summarise the results. The analysis findings were presented in tables.

### Ethical consideration

Ethical clearance was sought from the Research and Publications Committee of Muhimbili University of Health and Allied Sciences. The permission to conduct the study was sought from the authority of MNH. Written informed consents were obtained from the patients in accordance with the Declaration of Helsinki before recruitment.

## Results

**Figure 1.**
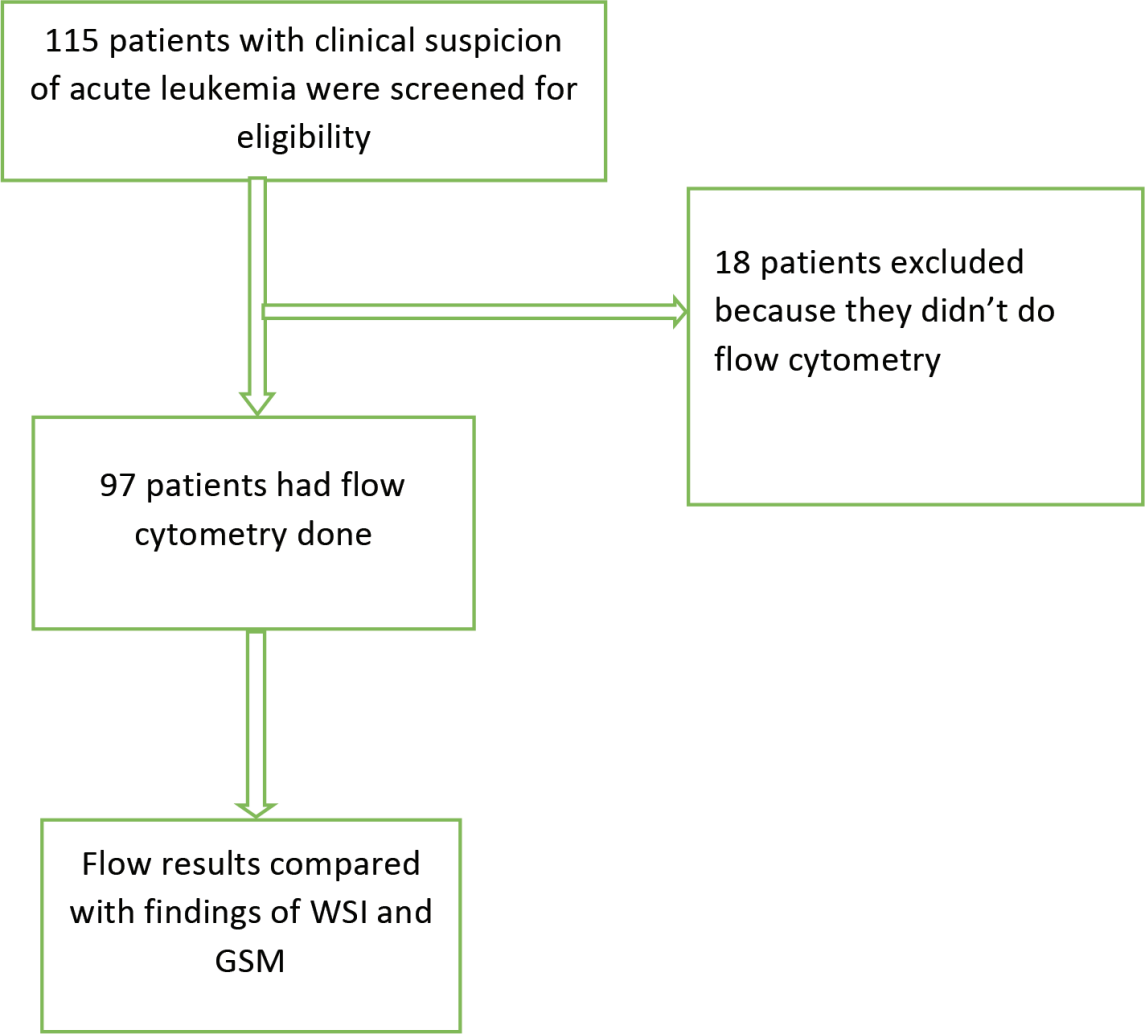
Flow chart of recruited patients and evaluated samples.

A total of 115 patients with clinical suspicion of AL were screened for eligibility; resulting in the exclusion of 18 patients who had not undergone flow cytometry testing. Out of the 97 remaining patients, 47 were confirmed to have AL, of which AML and ALL were 19 (40.4%) and 28 (59.6%) patients, respectively. The GSM and WSI of these patients were evaluated for the diagnosis of AL and its types (Figure 1).

Generally, GSM demonstrated high sensitivity (89.4%) and specificity (90.0%) in diagnosing AL, achieving an accuracy rate of 87.9% (Table 1). However, when distinguishing AML from ALL, GSM sensitivity decreased to 57.9%, while specificity remained high at 89.7%. GSM correctly identified only 11 out of 19 patients with AML. Conversely, in identifying ALL, GSM performed better, accurately identifying 21 out of 28 patients, resulting in a sensitivity of 75% and specificity of 91.3%.

In contrast, when compared to flow cytometry results, WSI accurately diagnosed AL in 38 out of 47 patients, demonstrating a reasonable sensitivity of 80.9% (Table 2). Notably, WSI exhibited high specificity (98%) and a low FP rate of only 2%. However, WSI showed lower sensitivity in differentiating AML (57.9%) and ALL (46.4%), while maintaining a specificity of 91% for both types.

## Discussion

In this study, our aim was to assess the performances of GSM, a commonly used technique for diagnosing AL and WSI which is a potential solution for overcoming the shortage of hematologists in resource-limited settings like Tanzania. We identified 47 patients with AL through flow cytometry, including 19 with AML and 28 with ALL. Our results indicate that GSM demonstrates reasonably high sensitivity and accuracy, around 90%, in diagnosing AL. This suggests that morphology is useful for the initial diagnosis, as most bone marrow aspirates at presentation have blasts exceeding 90%, making immature cells easily detectable under a microscope. However, up to 10% of cases may be missed or misclassified as non-AL, particularly when the blast percentage is not markedly high or blasts are not remarkably immature. While this underscores the importance of combining both morphology and flow cytometry when evaluating suspected AL cases [6], it also reinforces the value of GSM as a primary diagnostic tool for AL in resource-limited settings.

Similarly, WSI performance showed acceptable sensitivity (81%) and accuracy (90%), consistent with findings from other studies validating its use in diagnosing various malignancies [7, 8]. WSI was found to be non-inferior to conventional light microscopy [8, 9], suggesting its potential for replacing GSM in regions lacking hematologists, thereby fostering institutional collaborations for early referral of the patients. Laboratory personnel from peripheral centers without access to hematology consultations could collaborate with hematologists from tertiary centers in Tanzania to accurately screen patients likely to have AL and refer them for definitive diagnosis and timely treatment, thus potentially overcoming human resource shortages and shorten the time to diagnosis, which is crucial and potentially lifesaving in patients with AL.

However, both GSM and WSI faced challenges in distinguishing between AL types, with sensitivity ranging from 50% to 60%. Morphologically, AML blasts may resemble ALL, especially in undifferentiated and minimally differentiated subtypes, where granules may be minimally present or completely absent [6]. This indicates that morphology alone may not suffice for confidently classifying AL types for treatment purposes. Therefore, in resource-limited settings, where the cost of flow cytometry is prohibitive for most, institutions should strive to at least incorporate immunocytochemistry and immunohistochemistry markers in the morphological evaluation of AL. These stains can assist in lineage assignment for difficult AL cases where simple morphological assessment of blasts proves challenging [10, 11].

While advocating for and supporting advanced treatment options like the stem cell transplant services introduced under government sponsorship in Tanzania in 2023 [12], policymakers and the government should also prioritise enhancing other advanced diagnostic services. These include the routine availability of flow cytometry, cytogenetics and molecular studies at the tertiary centers, which are essential for accurate diagnosis and prognosis of AL.

Despite our study having demonstrated the utility of WSI in diagnosing AL, there are still pertinent research gaps that must be addressed in future studies to fully realise the potential of WSI in overcoming the challenge of late diagnosis, a common problem in many low and middle income countries (LMICs). In this study, bone marrow slides were used for diagnosing AL by WSI, as they maximised the visualization of blasts. However, to achieve the ultimate goal of shortening the time to diagnosis in clinical practice, it is crucial to evaluate the performance of WSI in peripheral blood, where fewer blasts may pose a diagnostic challenge. Additionally, in this study, a single experienced hematologist interpreted both GSM and WSI, eliminating the opportunity to assess inter-observer reliability of WSI among personnel with varying levels of expertise.

In conclusion, WSI is non-inferior to GSM, the mainstay for diagnosing AL in many LMICs. Therefore, WSI is a potential solution to overcome the challenges of late diagnosis of AL, which is often due to the limited number of hematologists and pathologists in peripheral centers.

## Conflicts of interest

The authors declare no conflicts of interest.

## Author contributions

HI – contributed in conception and designing of the work, data collection, drafting of the original manuscript, review and approval of the final draft; AN – contributed in data interpretation and analysis, drafting of the original manuscript, review and approval of the final draft,

AH – contributed in design of the work, data analysis, review and approval of the final draft; AS – contributed in conception and designing of the work, data interpretation, reviewing and approval of the final draft; CC – contributed in conception and designing of the work, data interpretation, reviewing and approval of the final draft.

## Figures and Tables

**Table 1.** Sensitivity and specificity of GSM compared to flow cytometry (gold standard) in the diagnosis of AL and its types.

		Flow cytometry *N* (%)		Sensitivity (%)	Specificity (%)	PPV (%)	NPV (%)	Accuracy (%)
		AL	No AL					
GSM *N* (%)	AL No AL	42 (89.4) 5 (10.6)	5 (10.0) 45 (90.0)	89.4	90.0	89.4	90.0	89.7
		AML	No AML					
	AML No AML	11 (57.9) 8 (42.1)	8 (10.3) 70 (89.7)	57.9	89.7	57.9	89.7	83.5
		ALL	No ALL					
	ALL No ALL	21 (75.0) 7 (25.0)	6 (8.7) 63 (91.3)	75.0	91.3	77.8	90.0	86.6

**Table 2.** Sensitivity and specificity of WSI compared to flow cytometry (gold standard) in the diagnosis of AL and its types.

		Flow cytometry *N* (%)		Sensitivity (%)	Specificity (%)	PPV (%)	NPV (%)	Accuracy (%)
		AL	No AL					
WSI *N* (%)	AL No AL	38 (80.9) 9 (19.1)	1 (2.0) 49 (98.0)	80.9	98.0	97.4	84.5	89.7
		AML	No AML					
	AML No AML	11 (57.9) 8 (42.1)	7 (9.0) 71 (91.0)	57.9	91.0	57.9	91.0	84.5
		ALL	No ALL					
	ALL No ALL	13 (46.4) 15 (53.6)	6 (8.7) 63 (91.3)	46.4	91.3	68.4	80.8	78.4
